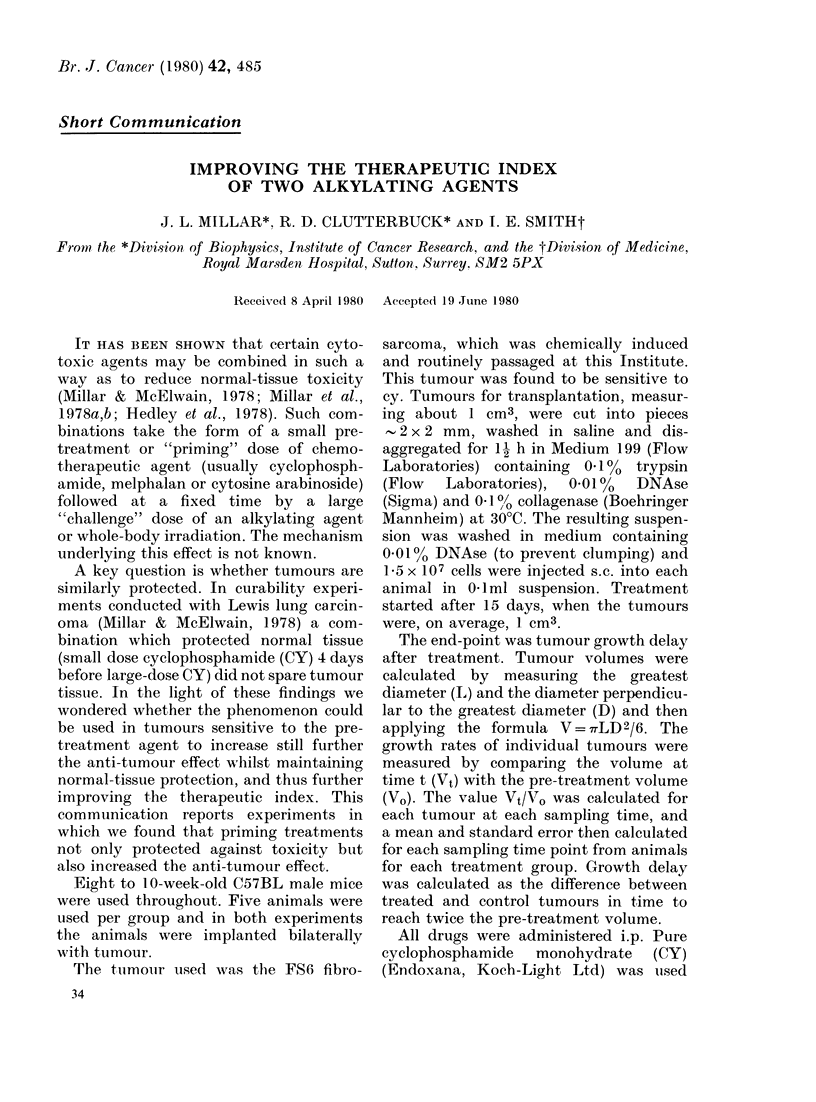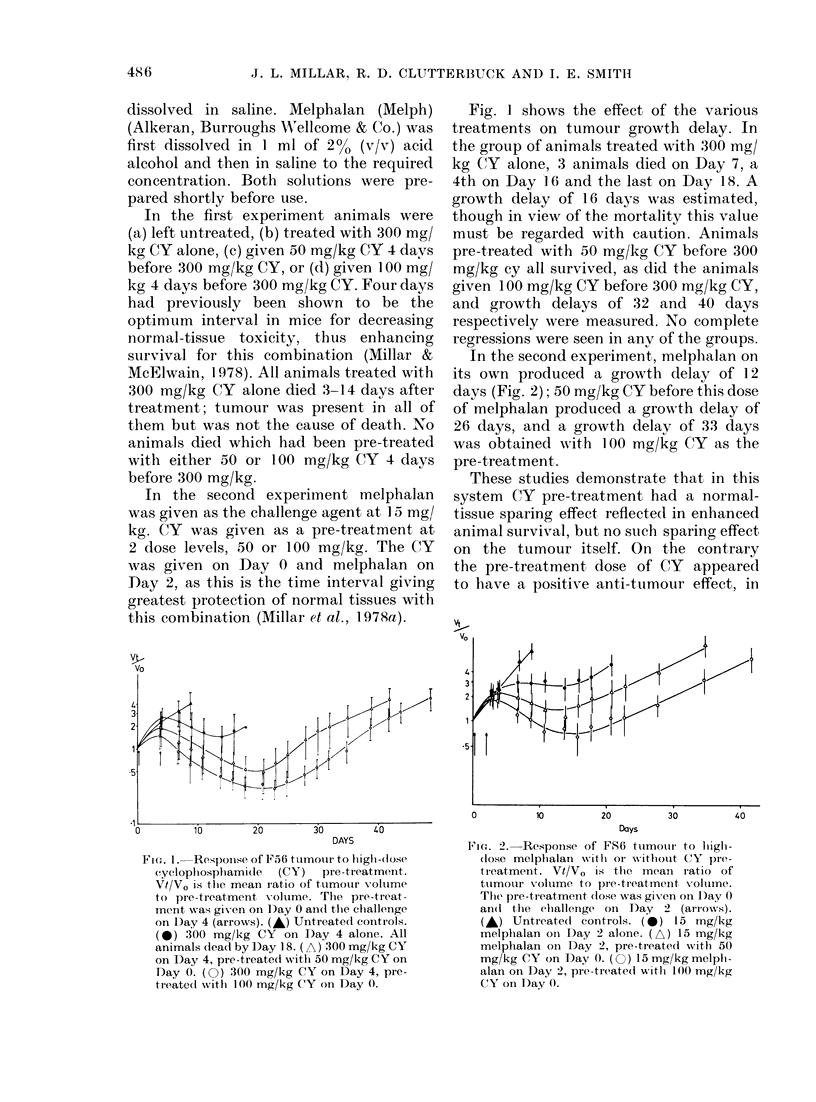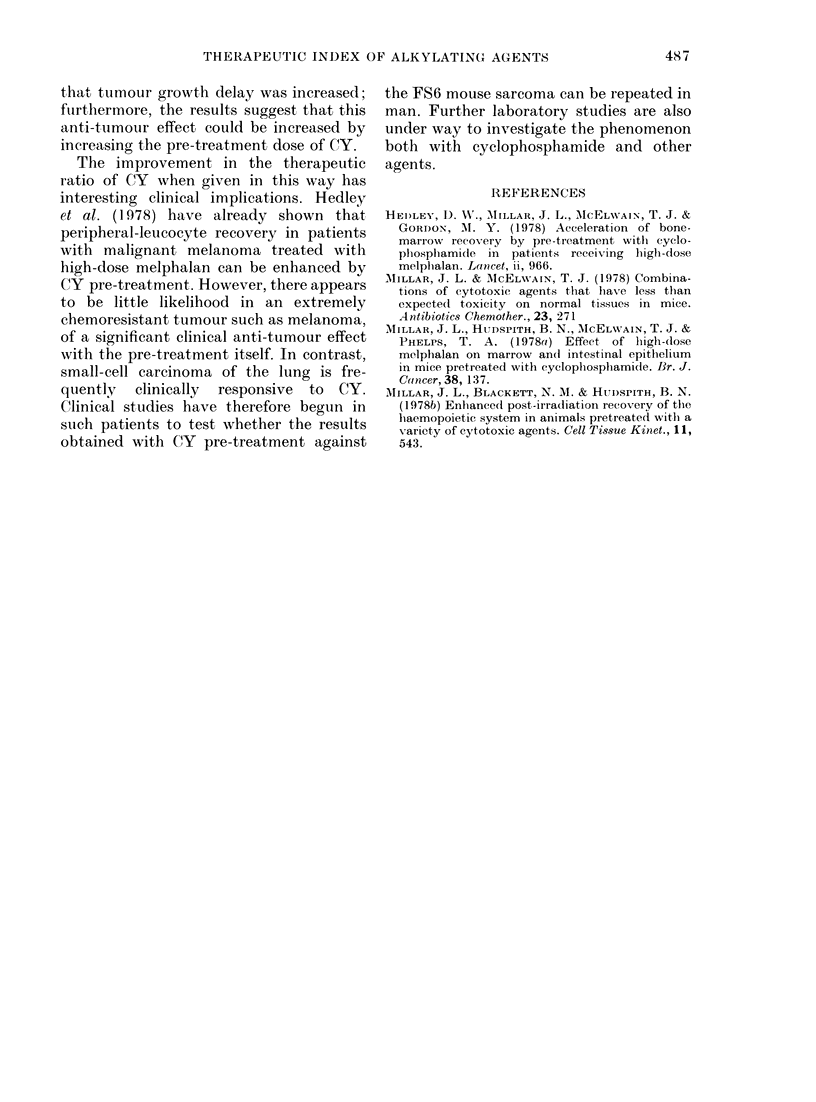# Improving the therapeutic index of two alkylating agents.

**DOI:** 10.1038/bjc.1980.263

**Published:** 1980-09

**Authors:** J. L. Millar, R. D. Clutterbuck, I. E. Smith


*Br. J. Cancer* (1980) **42**, 485

***Short Communication***

### IMPROVING THE THERAPEUTIC INDEX OF TWO ALKYLATING AGENTS

J. L. MILLAR*, R. D. CLUTTERBUCK* AND I. E. SMITH†

*From the *Division of Biophysics, Institute of Cancer Research, and the †Division of Medicine, Royal Marsden Hospital, Sutton, Surrey, SM2 5PX*

Received 8 April 1980 Accepted 19 June 1980

IT HAS BEEN SHOWN that certain cytotoxic agents may be combined in such a way as to reduce normal-tissue toxicity (Millar & McElwain, 1978; Millar *et al.*, 1978*a,b*; Hedley *et al.*, 1978). Such combinations take the form of a small pretreatment or "priming" dose of chemotherapeutic agent (usually cyclophosphamide, melphalan or cytosine arabinoside) followed at a fixed time by a large "challenge" dose of an alkylating agent or whole-body irradiation. The mechanism underlying this effect is not known.

A key question is whether tumours are similarly protected. In curability experiments conducted with Lewis lung carcinoma (Millar & McElwain, 1978) a combination which protected normal tissue (small dose cyclophosphamide (CY) 4 days before large-dose CY) did not spare tumour tissue. In the light of these findings we wondered whether the phenomenon could be used in tumours sensitive to the pretreatment agent to increase still further the anti-tumour effect whilst maintaining normal-tissue protection, and thus further improving the therapeutic index. This communication reports experiments in which we found that priming treatments not only protected against toxicity but also increased the anti-tumour effect.

Eight to 10-week-old C57BL male mice were used throughout. Five animals were used per group and in both experiments the animals were implanted bilaterally with tumour.

The tumour used was the FS6 fibrosarcoma, which was chemically induced and routinely passaged at this Institute. This tumour was found to be sensitive to cy. Tumours for transplantation, measuring about 1 $cm^3$, were cut into pieces $\sim 2 \times 2$ mm, washed in saline and disaggregated for $1\frac{1}{2}$ h in Medium 199 (Flow Laboratories) containing 0·1% trypsin (Flow Laboratories), 0·01% DNAse (Sigma) and 0·1% collagenase (Boehringer Mannheim) at 30°C. The resulting suspension was washed in medium containing 0·01% DNAse (to prevent clumping) and $1 \cdot 5 \times 10^7$ cells were injected s.c. into each animal in 0·1ml suspension. Treatment started after 15 days, when the tumours were, on average, 1 $cm^3$.

The end-point was tumour growth delay after treatment. Tumour volumes were calculated by measuring the greatest diameter (L) and the diameter perpendicular to the greatest diameter (D) and then applying the formula $V = \pi LD^2/6$. The growth rates of individual tumours were measured by comparing the volume at time t ($V_t$) with the pre-treatment volume ($V_0$). The value $V_t/V_0$ was calculated for each tumour at each sampling time, and a mean and standard error then calculated for each sampling time point from animals for each treatment group. Growth delay was calculated as the difference between treated and control tumours in time to reach twice the pre-treatment volume.

All drugs were administered i.p. Pure cyclophosphamide monohydrate (CY) (Endoxana, Koch-Light Ltd) was used dissolved in saline. Melphalan (Melph) (Alkeran, Burroughs Wellcome & Co.) was first dissolved in 1 ml of 2% (v/v) acid alcohol and then in saline to the required concentration. Both solutions were prepared shortly before use.

In the first experiment animals were (a) left untreated, (b) treated with 300 mg/kg CY alone, (c) given 50 mg/kg CY 4 days before 300 mg/kg CY, or (d) given 100 mg/kg 4 days before 300 mg/kg CY. Four days had previously been shown to be the optimum interval in mice for decreasing normal-tissue toxicity, thus enhancing survival for this combination (Millar & McElwain, 1978). All animals treated with 300 mg/kg CY alone died 3–14 days after treatment; tumour was present in all of them but was not the cause of death. No animals died which had been pre-treated with either 50 or 100 mg/kg CY 4 days before 300 mg/kg.

In the second experiment melphalan was given as the challenge agent at 15 mg/kg. CY was given as a pre-treatment at 2 dose levels, 50 or 100 mg/kg. The CY was given on Day 0 and melphalan on Day 2, as this is the time interval giving greatest protection of normal tissues with this combination (Millar *et al.*, 1978*a*).

FIG. 1.—Response of F56 tumour to high-dose cyclophosphamide (CY) pre-treatment. $Vt/V_0$ is the mean ratio of tumour volume to pre-treatment volume. The pre-treatment was given on Day 0 and the challenge on Day 4 (arrows). (▲) Untreated controls. (●) 300 mg/kg CY on Day 4 alone. All animals dead by Day 18. (△) 300 mg/kg CY on Day 4, pre-treated with 50 mg/kg CY on Day 0. (○) 300 mg/kg CY on Day 4, pre-treated with 100 mg/kg CY on Day 0.

Fig. 1 shows the effect of the various treatments on tumour growth delay. In the group of animals treated with 300 mg/kg CY alone, 3 animals died on Day 7, a 4th on Day 16 and the last on Day 18. A growth delay of 16 days was estimated, though in view of the mortality this value must be regarded with caution. Animals pre-treated with 50 mg/kg CY before 300 mg/kg cy all survived, as did the animals given 100 mg/kg CY before 300 mg/kg CY, and growth delays of 32 and 40 days respectively were measured. No complete regressions were seen in any of the groups.

In the second experiment, melphalan on its own produced a growth delay of 12 days (Fig. 2); 50 mg/kg CY before this dose of melphalan produced a growth delay of 26 days, and a growth delay of 33 days was obtained with 100 mg/kg CY as the pre-treatment.

These studies demonstrate that in this system CY pre-treatment had a normal-tissue sparing effect reflected in enhanced animal survival, but no such sparing effect on the tumour itself. On the contrary the pre-treatment dose of CY appeared to have a positive anti-tumour effect, in

FIG. 2.—Response of FS6 tumour to high-dose melphalan with or without CY pre-treatment. $Vt/V_0$ is the mean ratio of tumour volume to pre-treatment volume. The pre-treatment dose was given on Day 0 and the challenge on Day 2 (arrows). (▲) Untreated controls. (●) 15 mg/kg melphalan on Day 2 alone. (△) 15 mg/kg melphalan on Day 2, pre-treated with 50 mg/kg CY on Day 0. (○) 15 mg/kg melphalan on Day 2, pre-treated with 100 mg/kg CY on Day 0.

that tumour growth delay was increased; furthermore, the results suggest that this anti-tumour effect could be increased by increasing the pre-treatment dose of CY.

The improvement in the therapeutic ratio of CY when given in this way has interesting clinical implications. Hedley *et al.* (1978) have already shown that peripheral-leucocyte recovery in patients with malignant melanoma treated with high-dose melphalan can be enhanced by CY pre-treatment. However, there appears to be little likelihood in an extremely chemoresistant tumour such as melanoma, of a significant clinical anti-tumour effect with the pre-treatment itself. In contrast, small-cell carcinoma of the lung is frequently clinically responsive to CY. Clinical studies have therefore begun in such patients to test whether the results obtained with CY pre-treatment against the FS6 mouse sarcoma can be repeated in man. Further laboratory studies are also under way to investigate the phenomenon both with cyclophosphamide and other agents.

### REFERENCES

HEDLEY, D. W., MILLAR, J. L., McELWAIN, T. J. & GORDON, M. Y. (1978) Acceleration of bone-marrow recovery by pre-treatment with cyclophosphamide in patients receiving high-dose melphalan. *Lancet*, ii, 966.

MILLAR, J. L. & McELWAIN, T. J. (1978) Combinations of cytotoxic agents that have less than expected toxicity on normal tissues in mice. *Antibiotics Chemother.*, **23**, 271

MILLAR, J. L., HUDSPITH, B. N., McELWAIN, T. J. & PHELPS, T. A. (1978*a*) Effect of high-dose melphalan on marrow and intestinal epithelium in mice pretreated with cyclophosphamide. *Br. J. Cancer*, **38**, 137.

MILLAR, J. L., BLACKETT, N. M. & HUDSPITH, B. N. (1978*b*) Enhanced post-irradiation recovery of the haemopoietic system in animals pretreated with a variety of cytotoxic agents. *Cell Tissue Kinet.*, **11**, 543.